# Competition for acoustic space in a temperate-forest bird community

**DOI:** 10.1093/beheco/arad075

**Published:** 2023-09-23

**Authors:** Agata Staniewicz, Emilia Sokołowska, Adrianna Muszyńska, Michał Budka

**Affiliations:** Department of Behavioural Ecology, Adam Mickiewicz University in Poznań, Uniwersytetu Poznańskiego 6, 61-614 Poznań, Poland; Department of Behavioural Ecology, Adam Mickiewicz University in Poznań, Uniwersytetu Poznańskiego 6, 61-614 Poznań, Poland; Department of Behavioural Ecology, Adam Mickiewicz University in Poznań, Uniwersytetu Poznańskiego 6, 61-614 Poznań, Poland; Department of Behavioural Ecology, Adam Mickiewicz University in Poznań, Uniwersytetu Poznańskiego 6, 61-614 Poznań, Poland

**Keywords:** acoustic dissimilarity index, birdsong, interspecific competition, song dissimilarity index, song similarity

## Abstract

Animals that communicate by acoustic signaling share a common acoustic environment. Birds are particularly vocal examples, using a wide repertoire of broadcast signals for mate attraction and territorial defense. However, interference caused by sounds that overlap in frequency and time can disrupt signal detection and reduce reproductive success. Here, we investigated competition avoidance mechanisms used by the bird community inhabiting a primeval lowland temperate forest in Białowieża, Eastern Poland. We recorded the dawn chorus at 84 locations in early and late spring and calculated dissimilarity indices of the broadcast signals to examine how species with greater song similarities use spatial and temporal partitioning to avoid competition for acoustic space throughout the breeding season. The bird community changed its use of acoustic space throughout the day and season. Birds did not use spatial partitioning of signal space when we looked at recording locations over the whole study period, but they did in a seasonal context, with species more acoustically different than expected by chance recorded at the same point in the same part of the season. Our results also indicate that daily temporal niche partitioning may only occur at certain times before sunrise, with no evidence of large-scale temporal partitioning between species vocalizing during the same 1-min recordings in daytime. These results contribute toward our understanding of the evolution of bird communication and highlight the strategies employed by different species to improve their signal transmission.

## Teaser

To improve communication, birds use flexible approaches to separate their vocal activity in space and time. Similarly, singing species occupy different forest locations in different times of the year, combining spatial and seasonal partitioning of signal space. Over the course of the day, temporal niche separation is dependent on the time of day, with acoustically different species singing together before sunrise, whereas species active together in daylight hours are more acoustically similar.

## INTRODUCTION

Acoustic communication has evolved in many animal taxa, including mammals, birds, reptiles, amphibians, fish, and insects. All the vocalizing animals inhabiting the same environment share the common acoustic space. Each species occupies a specific region of this signal space, determined by the parameters of its vocalizations, including time, frequency range, and amplitude (Nelson and Marler 1990; Chek et al. 2003). The frequency range is correlated with the size and shape of the vocal organ (Podos 2001; Fletcher 2004; Riede et al. 2006), with larger animals generally producing lower frequency sounds which can propagate further (Marten et al. 1977; Ophir et al. 2010; Mikula et al. 2021). The amplitude defines the amount of energy contained in the signal. Louder sounds contain more energy, and increasing the amplitude can mitigate the masking effects of ambient noise (Brumm 2004; Nemeth et al. 2013). The time when the animal vocalizes—both on seasonal and daily scales—as well as the duration of the signal constitute the temporal parameters of the signal space and depend on the biology and ecology of the species, as well as the economics of communication.

Sounds that overlap in frequency and time result in acoustic interference, which can mask or alter some parts of the signal, limit signal detection, and—particularly in the case of broadcast signals—can lead to lower reproductive success or survival (Bradbury and Vehrencamp 2011). In natural communities, interspecific competition for acoustic signal space can occur between closely related species within, for example, birds (Planqué and Slabbekoorn 2008; Kirschel et al. 2009; Luther 2009; Krishnan and Tamma 2016; Bolanos-Sittler et al. 2021), or anurans (Hodl 1977; Narins 1995; Chek et al 2003; Allen-Ankins and Schwarzkopf 2021; Sugai et al. 2021), as well as between phylogenetically divergent taxonomic groups such as birds and cicadas (Hart et al. 2015; Stanley et al. 2016), or frogs and insects (Greenfield 1994). Noise can also reduce acoustic space available to animals, leading to competition avoidance. Anthropogenic noise has been widely documented to cause shifts in signal space of birds (Gil et al. 2015; De Framond and Brumm 2022), frogs (Parris et al. 2009), and marine mammals (Nowacek et al. 2007), while abiotic noise such ocean surf, river, and stream noise modified the vocalizing behavior of birds and frogs (Zhao et al. 2018; Reed et al. 2022).

Interspecific competition can be reduced through signal space partitioning and can occur along several axes. Species may broadcast from different physical locations, such as microhabitats or perch height (Nemeth et al. 2002; Diwakar and Balakrishnan 2007; Jain and Balakrishnan 2012; Lima et al. 2019; Chitnis et al. 2020). Temporal partitioning can also occur, with species vocalizing in different seasons, times of day (Luther 2008), or in gaps between other species’ vocalizations (Cody and Brown 1969; Brumm 2006; Bleach et al. 2015; Herrick et al. 2018). Coexisting species may also evolve divergent spectral frequencies (Both and Grant 2012; Villanueva-Rivera 2014; Krishnan and Tamma 2016) or shift their signal frequency in response to other sounds broadcast in the area (Lopez et al. 1988).

Birds are a particularly vocal taxonomic group, with acoustic signals being key to mate attraction and territory defense during the breeding season for many species. Many of the studies examining competition for acoustic space in birds to date have focused mainly on individual or small numbers of species (Popp et al. 1985; Brumm 2006; Kirschel et al. 2009), primarily in the tropics (Luther 2009; Bolanos-Sittler et al. 2021; Hart et al. 2021). Studies on large-scale, community-level trends in bird signal space partitioning have primarily focused on tropical bird communities (Luther 2009; Krishnan 2019; Hart et al. 2021; Lahiri et al. 2021), with comparatively little focus placed on temperate bird communities (Chhaya et al. 2021), which face the additional pressure of a short, synchronized breeding season, potentially increasing the pressure on acoustic space. To fully understand the evolution of acoustic signals, we should consider how species adapt their signal characteristics not only to the physical conditions of the environment shaped by vegetation, temperature, or humidity but also to the acoustic properties of their environment created by anthrophonic, geophonic, and biophonic sounds. In particular, the effects of interspecific acoustic interactions on the evolution of acoustic signals are poorly known.

Here, we investigated the mechanisms used to reduce competition for acoustic space among species in a complex bird community inhabiting Białowieża Forest—a relatively natural temperate ecosystem with low noise pollution and acoustic space dominated by birds. We focused on the broadcast signals used in mate attraction and territory defense, which we here refer to as songs in both passerine and non-passerine species. We calculated song dissimilarity indices (SDI), based on the Acoustic Dissimilarity Index (Sueur et al. 2008), for birds in a temperate forest and tested whether species with spectrally similar songs occur in different areas (spatial partitioning), or if they avoid competition by singing at different times of season or day (temporal partitioning). Effective acoustic communication is one of the most important factors ensuring survival and reproductive success for animals such as birds, therefore to minimize competition for acoustic space, we predict that coexisting or co-signaling species exhibit more dissimilar vocalizations than those that inhabit spatially separate areas or vocalize at different parts of a season or day.

## METHODS

### Study site

We carried out the study in Białowieża Forest—a large lowland forest complex encompassing ca 1450 km^2^, which spreads across the Polish-Belarusian border. Our study area was in the south-eastern part of the Polish section of the forest complex—an area within the territorial range of the Białowieża Forest District (120 km^2^, ca 20% of the Polish part of the Białowieża Forest). The study site covered areas under forest management (65%) as well as protected forests in nature reserves (35%). The average forest age in Białowieża Forest District is estimated to be 90 years, and the forests older than 140 years cover more than 27% of the area. The dominant tree species are spruce (32%), pine (20%), oak (20%), maple (19%), and birch (6%). The study site is characterized by several unique features: huge variability of forest types (dry, wet, coniferous, mixed, deciduous), multi-story profile of stands, diverse plant communities, large amount of deadwood and uprooted trees, and high abundance of tree holes. The Białowieża Forest houses many animal species that are rare or extinct elsewhere in Europe, including European bison *(Bison bonasus)*, wolf *(Canis lupus)*, lynx (*Lynx lynx*), and more than 150 breeding bird species. The climate in Białowieża Forest is subcontinental, with the average annual temperature of 7.3 °C (from 5.9 to 9.2 °C) and precipitation of 625 mm (data for the period from 1985 to 2015; Boczoń et al. 2018). The breeding season for most bird species comprises the beginning of April to the end of June (Wesołowski et al. 2015).

### Dawn chorus recording

We randomly selected 84 recording points within the territorial range of the Białowieża Forest District (see Budka et al. 2023 for exact locations of recording points). First, we generated regular 1 × 1 km grid; then, we removed points that were located less than 500 m from the main roads and buildings, or which were located less than 100 m from the forest edge. From the remaining points, we selected 84 for the dawn chorus recording. Our recording points were located both in protected and unprotected forests. The random distribution of recording points should reflect the variety of forest types, age, and classes, as well as water content in the study site. The large distance between neighboring points (from 470 to 1180 m) ensured that we did not record the same individuals from different recording points.

We recorded the dawn chorus twice at each recording point: during early (from 20 April to 2 May 2021) and late survey (from 18 May to 26 May 2021). Throughout each survey, we collected 6 h of dawn chorus recordings—from 2 h before sunrise to 4 h after sunrise (sunrise: April 20 = 05:15; May 18 = 04:23; local time). Such seasonal and daily distribution of surveys enables the detection of both early and late breeding species, as well as species active during the day and at night.

We used 10 Song Meter SM3 acoustic recorders (Wildlife Acoustics) with a single built-in omnidirectional microphone SMM-A1 (sensitivity −11 ± 4 dB; signal-to-noise ratio > 68 dB) to record the dawn chorus. Before the recording, we tested each microphone by using a sound level calibrator (VOLTCRAFT SLC-100). The calibrator generated a 94dB SPL 1kHz tone. The microphone passed the test when the dB level was higher than −16 dB in Song Meter Mini “test microphone window” (according to the producer’s recommendation). This procedure allowed us to maintain a relatively constant sensitivity of the microphones used in the study. We applied the same recording settings across the study (16-bit wav file format, 48 kHz sampling rate, low- and high-frequency filters off, gain 24 dB). Recorders were placed on trees, 8 m above ground level, on the northern aspect of the tree, and with the microphone pointing west. When we met difficulties in finding an appropriate tree, or when the randomly chosen point was located on a footpath, we moved the recording point, but no more than 100 m from the original location. We collected all dawn chorus recordings in good weather conditions (no strong wind or heavy rain). The autonomous recorder detection range is species-specific and depends on the frequency and amplitude of the vocalization, but for most songbirds it should range between 100 and 150 m (Yip et al. 2017).

### Acoustic analysis

During each survey, we analyzed 36 one-minute sound samples per recording site (1 min every 10 min of the dawn chorus recording, beginning from 2 h before sunrise to 4 h after sunrise). Sound samples were analyzed by manual spectrogram scanning and listening to recordings by three observers (A.M., E.S., and M.B.). We randomly assigned sound samples to observers, with each observer analyzing one-third of the data. One observer analyzed all recordings from one survey at recording point. We used Raven Pro 1.6.1 software (Cornell Laboratory of Ornithology, Ithaca, NY, USA) with the following settings to analyze sound samples: window type = Hamming, window size = 23.1 ms, overlap = 75%.

In each 1-min sound sample, we identified all singing species. As a song we considered the broadcast signals—songs in songbirds and vocalizations of non-songbirds with the primary function of mate attraction and territory defense (e.g., territorial calls of owls, drumming of woodpeckers). Typical calls, which are simple, short, produced by both sexes across the year in particular context related to specific function like flight, threat, alarm, or feeding, were excluded from the analysis. When we had difficulties with species identification, we compared the problematic sound sample with publicly available examples, such as Xeno-canto birdsong database (https://www.xeno-canto.org). In total, we analyzed 6048 one-minute sound samples (36 one-minute samples × 84 recording points × 2 surveys per season) and prepared lists of singing species recorded in each 1-min sample. The 1-min sound samples used in the study are available in open data repository (see Budka et al. 2023).

### Song dissimilarity index

We calculated the SDI for each pair of the species present in Białowieża Forest using good quality recordings of 55 reference species from the area (8 ± 2 individuals per species, 414 individuals in total, the full list is given in the Supplementary Materials). The index is based on the Acoustic Dissimilarity Index developed by Sueur, Pavoine et al. (2008), which estimates both the temporal and spectral dissimilarities of two recordings. To obtain the song samples, we recorded songs of 62 individuals of 10 species in Białowieża Forest between 19–28 April 2021. The recordings were made using a digital recorder (Marantz PMD661) at a sampling rate of 48 kHz with16-bit accuracy, with a Sennheiser directional microphone. We supplemented these with recordings of 352 individuals downloaded from Xeno-canto, recorded at a minimum 44.1 kHz sampling rate. As the files from Xeno-canto were saved in mp3 format, we first converted our recordings to mp3 format using Audacity 3.1.3 software (https://audacityteam.org) and converted all mp3 files into wav format using “fix_wavs()” function in warbleR package (44.1 kHz sampling rate, 16-bit, mono file; Araya-Salas and Smith-Vidaurre 2017).

From each recording, which corresponded to each individual bird, we manually selected between 2 and 12 (mean = 7.17, SD = 2.40) songs that had no background noise using the spectrographic representation with the software RavenPro 1.6.1 (window = Hanning, FFT = 1024). For species with very long songs, we selected multiple 10 s portions of the song instead of individual songs. We applied bandpass filter to remove all noise above and below the frequency range of the songs from the file to ensure that only the signal of the song was used to compute the index. We extracted individual songs in R 4.1.2 (https://www.R-project.org) and calculated the spectral dissimilarity index for each pair of songs using the “diffspec()” function in the Seewave package (Sueur et al. 2008). To test how the file compression of the mp3 format affected the index results, we compared the index values of the same original and converted files recorded in Białowieża (original files mean = 0.513 ± 0.224, converted files mean = 0.517 ± 0.228; Cohen’s *D* = 0.473), which revealed a small to moderate effect of conversion. To reduce this effect, we used files converted from mp3 to wav in all calculations. We used one-way ANOVA to compare the resulting intraindividual, intraspecific, and interspecific index values.

### Statistical analysis

To examine the effect of song spectral dissimilarity on the singing species community composition, we performed three levels of analysis using R 4.1.2. To determine whether species occurring at the same location throughout the breeding season are less spectrally similar than a random composition of species recorded in the forest, for each recording point, we generated 1000 null model communities composed of a random selection of species which matched the number of species originally recorded at the point, as well as the overall proportion of the species recorded. We assigned the SDI value to each species pair in the observed and null model communities. As the values were not normally distributed, we used paired Wilcoxon signed rank tests to compare the dissimilarity index values of the species community observed at each point to the 1000 null models. We then calculated the proportion of null models with mean song dissimilarity indices, which were significantly higher, lower, or not different from those observed at each point (significance level *P* < 0.05).

To determine whether species occurring at the same location avoid acoustic competition by singing at different times of the season, we compiled the list of species present at each point during the early or late season and generated 1000 null model communities for each point in each season, matching the number of species originally recorded at the point, the overall proportion of the species recorded and the overall community composition for each season. We assigned the SDI value to each species pair in the observed and null model communities. We used paired Wilcoxon signed rank tests to compare the pooled mean dissimilarity index value of the species community observed at each of the 84 points to the 1000 pooled null model mean dissimilarity index values from the 84 points, and calculated the proportion of null model communities with mean song dissimilarity indices which were significantly higher, lower or not different to those observed at each point (significance level *P* < 0.05).

To determine whether species occurring at the same location avoid acoustic competition by singing at different times of day, we compiled the list of species present at each point during each of the 1-min recordings and removed the recordings with only one species present (*n* = 418) from the analysis. We generated 1000 null model communities for each recording, matching the number of species originally recorded in each minute and the overall community composition for each point on that day. To represent the common and rare species, we included the proportion of the recordings on the day (n/36) that a species was registered at the sampling point. We assigned the SDI value to each species pair in the observed and null model communities. We used paired Wilcoxon signed rank tests to compare the pooled mean dissimilarity index values of the species community observed at the 84 points to the 1000 pooled mean values of the null models from the 84 points and calculated the proportion of null models with mean song dissimilarity indices which were significantly higher, lower, or not different to those observed at each point (significance level *P* < 0.05). We then repeated this analysis separately on three 2-h sections of the day (2 h before sunrise, 2 h immediately after sunrise, and 3–4 h after sunrise) to determine if the light levels and time of day affected the species composition and competition for acoustic space. For each section of the day, we generated 1000 null models for each recording, matching the number of species originally recorded in each minute and the overall community composition for each point for that 2-h period. To represent the common and rare species, we included the proportion of the recordings in the 2-h period (n/12) that a species was registered at the sampling point. When generating all null models, we used the same seed using the “set.seed()” function in R, to ensure the reproducibility of the results.

Finally, to determine how the number of species detected affects their song dissimilarity and whether it changes throughout the day, we calculated the Spearman’s rank correlation coefficients for the mean number of species pairs detected with the mean SDI for each of the 36 one-minute recording samples throughout the day in the early and late surveys. To determine how the change in daylight affects the bird activity and their song dissimilarity, we calculated further Spearman’s rank correlation coefficients for the number of species pairs detected with the time of the 1-min recording throughout the day, the SDI with the time of 1-min recording throughout the day, and separately for the number of species pairs detected with the SDI before and after sunrise in 1-min recording samples in each of the two surveys.

## RESULTS

### Song dissimilarity index

**Figure 1:**
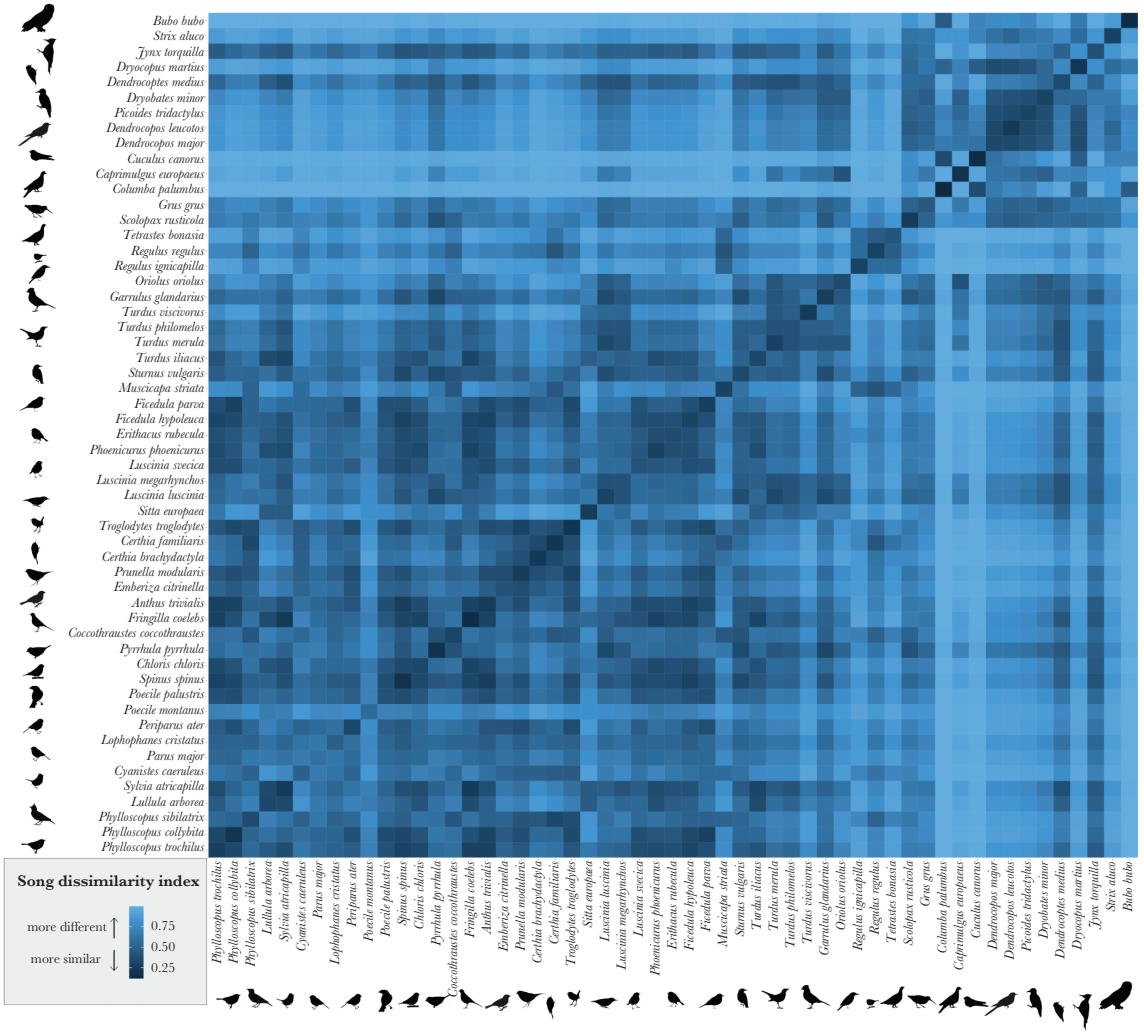
SDI values calculated for the 55 reference species from Białowieża Forest. Dark colors represent high similarity (low SDI value), and light colors represent low similarity (high SDI value). The diagonal line represents intraspecific comparisons.

**Figure 2:**
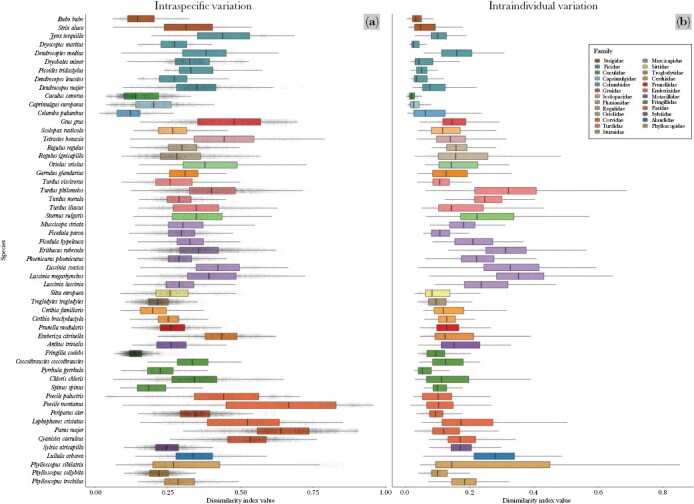
(a) Intraspecific and (b) intraindividual variation in SDI values for the 55 reference species. The boxes depict 25th percentiles, median line, and 75th percentiles of the measured values, and the whiskers represent 95% confidence intervals. The colors denote the family taxonomic groups.

The SDI value (0–1) represents the spectral dissimilarity of two recorded songs: low values indicate greater similarity and high values indicate greater dissimilarity between the songs. In total, we obtained the index values for 8 847 650 song comparisons (*n* songs = 2975, *n* individuals = 414, *n* species = 55, Figure 1). The index values between the species were significantly higher than within the species, and the index values within the species were significantly higher than within the individuals (*F* = 263 767, df = 2, 8 847 647, *P* < 0.001). The mean interspecific SDI value was 0.686 ± 0.213 (range from 0.074 to 0.999), the mean dissimilarity index value within the species was 0.348 ± 0.126 (range from 0.014 to 0.958), and the mean dissimilarity index value within individuals was 0.168 ± 0.123 (range from 0.003 to 0.853). The species varied significantly in their intraspecific SDI values (*F* = 4915.6, df = 2, 163 669, *P* < 0.001), ranging from species with mean intraspecific index values < 0.200 (Eurasian eagle-owl *Bubo bubo*, common wood pigeon *Columba palumbus*, common cuckoo *Cuculus canorus*, common chaffinch *Fringilla coelebes*, Eurasian siskin *Spinus spinus*), to species with mean intraspecific index value > 0.600 (great tit *Parus major*, willow tit *Poecille montanus*, Figure 2a). The intraindividual SDI value also varied significantly between the species (*F* = 361.46, df = 54, 20 679, *P* < 0.001), from species with mean intraindividual SDI value < 0.100 (Eurasian eagle-owl, European nightjar *Caprimulgus europaeus*, common cuckoo, white-backed woodpecker *Dendrocopos leucotos*, lesser-spotted woodpecker *Dryobates minor*, black woodpecker *Dryocopus martius*, Eurasian three-toed woodpecker *Picoides tridactylus*, Eurasian bullfinch *Pyrrhula pyrrhula*), to species with mean intraindividual index value > 0.300 (Common nightingale *Luscinia megarhynchos*, bluethroat *Luscinia svecica*, wood warbler *Phylloscopus sibilatrix*, song thrush *Turdus philomelos*, Figure 2b).

### Acoustic community composition in Białowieża Forest

**Figure 3:**
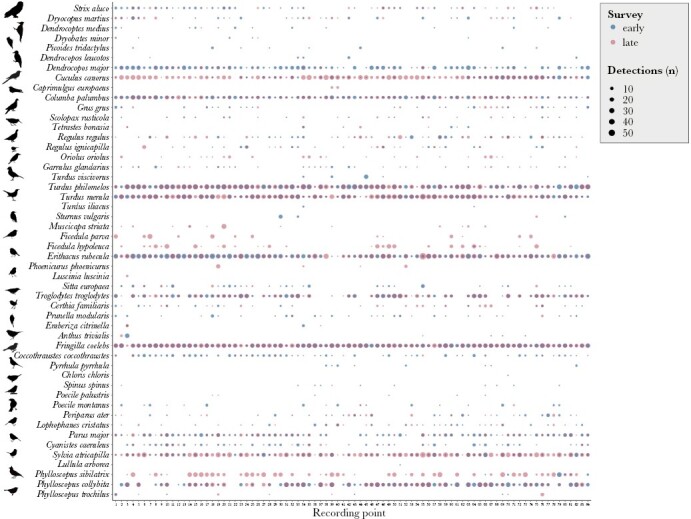
Frequency of species occurrence at each of the 84 recording points in Białowieża Forest during the early (blue) and late (pink) recording session.

We made 20743 species detections and recorded 65 bird species. On average, we recorded 20.8 ± 3.46 bird species per recording site (range from 15 to 31) and 15.3 ± 3.14 species per survey (range from 7 to 25). Five species (European robin *Erithacus rubecula*, common chaffinch, Eurasian blackcap *Sylvia atricapilla*, common blackbird *Turdus merula*, and song thrush) were recorded in all 84 recording sites, 16 species were recorded in more than 50% of recording sites while 29 species were recorded in less than 10% of recording sites (Figure 3). Fifteen species that were recorded in < 5 instances and/or were not typical forest inhabitants included in the original reference recording SDI calculation were removed from further analysis.

**Figure 4:**
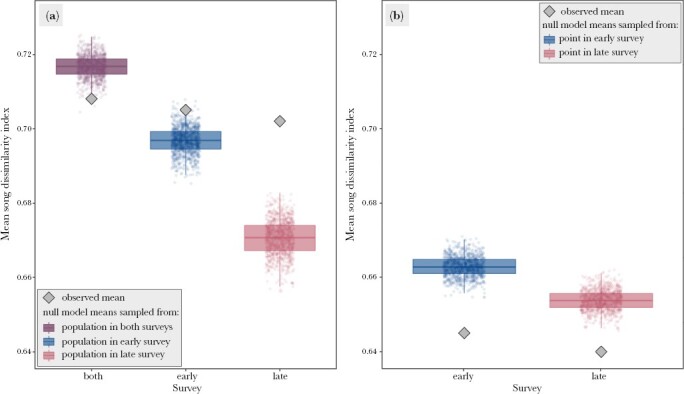
(a) The mean song dissimilarity index values of the observed (gray diamond) and the 1000 null model bird species communities at the same recording point throughout the study period (purple), in the early survey (blue) and the late survey (pink). The boxes depict 25th percentiles, median line, and 75th percentiles of the means of the null models, and the whiskers represent 95% confidence intervals. (b) The mean song dissimilarity index values of the observed (gray diamond) and the 1000 null model bird species communities in the 1-min recording sample, in the early survey (blue) and the late survey (pink). The boxes depict 25th percentiles, median line and 75th percentiles of the means of the null models, and the whiskers represent 95% confidence intervals.

The mean SDI per recording point throughout the duration of the study was 0.708 ± 0.021 (*n* = 84, range from 0.647 to 0.762), with 69% of the random communities scoring a significantly higher index value, and 31% of the random communities not being significantly different to the observed values. There were no random communities with significantly lower index values than those observed (Figure 4a).

During the early survey, the mean SDI for each point was 0.705 ± 0.028 (*n* = 84, range from 0.633 to 0.789), with 26.3% of the random communities scoring a significantly lower value, and 73.7% of the random communities not being significantly different. There were no random communities with significantly higher values. During the late survey, the mean SDI for each point was 0.702 ± 0.031 (*n* = 84, range from 0.560 to 0.752), with 100% of the random communities scoring a significantly lower index value than the observed one (Figure 4a).

During a single 1-min recoding, we identified 4.7 ± 1.71 species on average (range from 1 to 11). The mean SDI during each 1-min recording in the early season was 0.645 ± 0.133 (*n* = 2326, range from 0.240 to 0.999), with 99.9% of random communities scoring a significantly higher index value, and 0.1% of random communities not being significantly different to the observed value. In the late season, the mean index value during each 1-min recording was 0.640 ± 0.144 (*n* = 2465, range from 0.240 to 0.999), with 98.6% of random communities scoring a higher index value, and 1.4% of random communities not being significantly different to the observed value. In both surveys, there were no random communities with significantly lower index values than those observed (Figure 4b).

**Figure 5:**
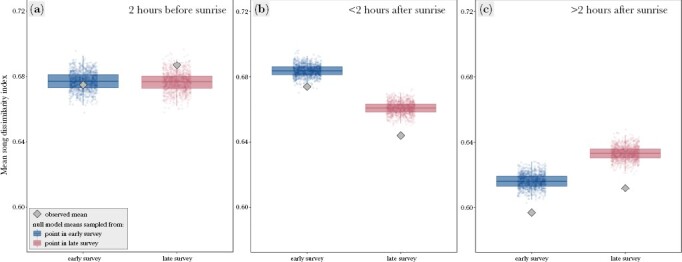
The mean song dissimilarity index values of the observed (gray diamond) and the 1000 null model bird species communities in the 1-min recording sample, in the early survey (blue) and the late survey (pink) at the same recording point during (a) the first 2 h before sunrise, (b) during the first 2 h after sunrise, (c) during the 3–4 h after sunrise. The boxes depict 25th percentiles, median line, and 75th percentiles of the means of the null models, and the whiskers represent 95% confidence intervals.

During the 2 h before sunrise, we identified 4.0 ± 1.66 species (range from 1 to 9) per recording sample in the early survey, and 4.1 ± 1.68 species (range from 1 to 11) in the late survey. The mean SDI during each 1-min recording before sunrise in the early survey was 0.675 ± 0.120 (*n* = 462, range from 0.330 to 0.990), with 0.6% of random communities scoring a significantly higher index value, 0.3% scoring a significantly lower index value and 99.1% not being significantly different to the observed value. During the late survey, the mean SDI was 0.687 ± 0.147 (range from 0.341 to 0.986), with 28.2% of the random communities scoring a significantly lower index value, and no random communities with significantly higher index value than observed (Figure 5a).

During the first 2 h after sunrise, we identified 5.1 ± 1.68 species (range from 1 to 10) per recording sample in the early survey, and 5.2 ± 1.66 species (range from 1 to 11) in the late survey. The mean SDI during each 1-min recording in the early survey was 0.674 ± 0.119 (*n* = 974, range from 0.350 to 0.999), with 43.8% of the random communities scoring a significantly higher index value, and no random communities with significantly lower index values. During the late survey, the mean SDI was 0.644 ± 0.135 (range from 0.240 to 0.999), with 99.1% of the random communities scoring a significantly higher index value, and no random communities with significantly lower index value than observed (Figure 5b).

During the third and fourth hours after sunrise, we identified 4.3 ± 1.59 species (range from 1 to 9) in the early survey and 4.7 ± 1.62 species (range from 1 to 9) in the late survey. The mean SDI during each 1-min recording in the early survey was 0.597 ± 0.140 (*n* = 974, range from 0.240 to 0.999), with 92.9% of the random communities scoring a significantly higher index value, and no random communities with significantly lower index values. During the late survey, the mean SDI was 0.612 ± 0.146 (range from 0.240 to 0.999), with 99.6% of the random communities scoring a significantly higher index value, and no random communities with significantly lower index value than observed (Figure 5c).

### Daily changes in bird song similarity

**Figure 6:**
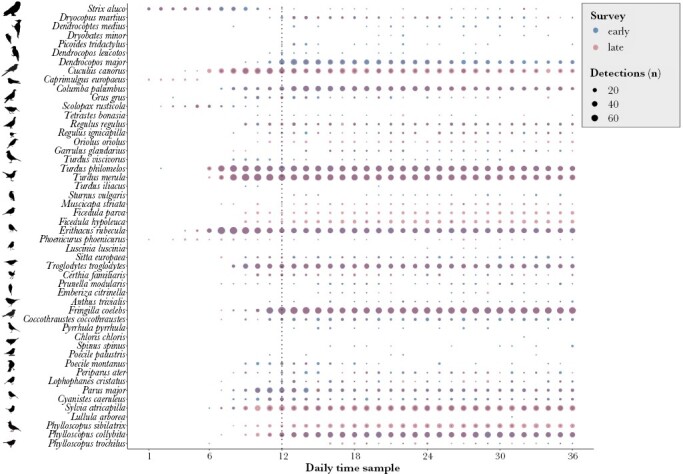
Frequency of species occurrence during each of the 36 one-minute recordings throughout the day in the early (blue) and late (pink) survey. The dotted vertical line marks the sunrise.

In both surveys, the species composition changed throughout the day. None of the species appeared on all 1-min recordings throughout the daily recording period, however, ten species (common wood pigeon, European robin, common chaffinch, great tit, common chiffchaff *Phylloscopus collybita*, wood warbler, Eurasian blackcap, Eurasian wren *Troglodytes troglodytes*, common blackbird, and song thrush), were recorded in all 1-min recording periods after sunrise in both surveys (Figure 6). Two species (European nightjar and Eurasian woodcock *Scolopax rusticola*) were recorded only before sunrise, and seven species appeared only on recordings after sunrise (European greenfinch *Chloris chloris*, lesser-spotted woodpecker, yellowhammer *Emberiza citrinella*, woodlark *Lullula arborea*, thrush nightingale *Luscinia luscinia*, Eurasian three-toed woodpecker, and Eurasian siskin).

**Figure 7:**
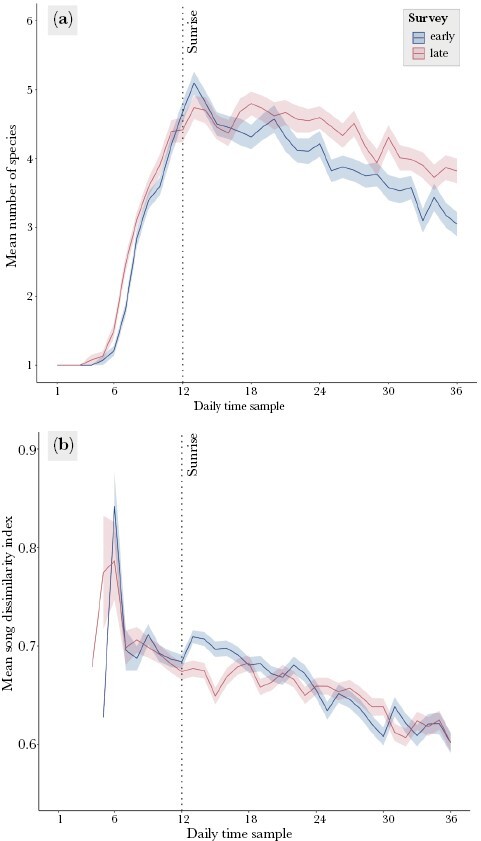
(a) Mean number of species recorded during each of the 36 one-minute recording samples throughout the day during the early (blue) and late (pink) surveys. (b) Mean song dissimilarity index values were calculated for the species pairs detected during each of the 36 one-minute recording samples throughout the day during the early (blue) and late (pink) surveys. The ribbons represent standard error. The dotted vertical line marks the sunrise.

The mean number of species pairs was significantly moderately negatively correlated with the time of day both during the early (ρ = −0.469, *P* < 0.001) and late survey (ρ = −0.315, *P* < 0.001), but this trend was affected by daylight, with a strong positive correlation between the mean number of species pairs and time of day before sunrise (both surveys: ρ = 1, *P* < 0.001), and a strong negative correlation between the number of species pairs and time of day after sunrise (early survey: ρ = −0.893, *P* < 0.001; late survey: ρ = −0.736, *P* < 0.001, Figure 7a). The mean SDI was also strongly negatively correlated with the time of day in both early (ρ = −0.915, *P* < 0.001) and late survey (ρ = −0.885, *P* < 0.001, Figure 7b).

Throughout the day during the early survey, there was a significant moderate positive correlation between the mean SDI and the mean number of species pairs detected on the 1-min recording sample (ρ = 0.572, *P* < 0.001). This correlation was also present in the late survey, although it was weaker (ρ = 0.337, *P* < 0.001). In both early and late survey, the mean SDI was strongly negatively correlated with the mean number of species pairs during the 2 h before sunrise (early survey: ρ = −0.700, *P* < 0.001; late survey: ρ = −0.932, *P* < 0.001), and strongly positively correlated during the 4 h after sunrise (early survey: ρ = 0.862, *P* < 0.001; late survey: ρ = 0.807, *P* < 0.001).

## DISCUSSION

The SDI captured the complexity of bird vocalizations, with low values reflecting greater acoustic similarity and higher values indicating greater dissimilarity between songs. Interspecific song comparisons provided higher SDI values than intraspecific comparisons, which were, in turn, higher than intraindividual song comparisons. Furthermore, species with more variable song repertoires, such as members of the tit family (Paridae), had higher intraspecific SDI values than species with conserved songs like those of the common wood pigeon or common chaffinch. Yet, species with high intraspecific SDI did not necessarily have high intraindividual SDI values. For example, the repertoires of individual great tits usually consist of two to six stereotypical song types, but they vary even between the males found in the same location (McGregor and Krebs 1982; Franco and Slabbekoorn 2009). The great tits in our study had a very high intraspecific SDI (0.642 ± 0.123), but the intraindividual differences were relatively low (0.154 ± 0.128), reflecting the repertoire diversity within the species and the limited number of song types produced by a bird in a single recording. Intraindividual SDI values also varied between species, from low in birds with typically short, single-type stereotypically produced songs such as those of the common cuckoo or the drumming of several of the woodpecker species, to high SDI values in species producing long, individually variable, and complex songs as recorded in the common nightingale, song thrush or members of the flycatcher family (Muscicapidae).

Using the SDI, our results show that the bird community in Białowieża Forest changed its use of acoustic space throughout the day and season. While the vocalizations of species recorded at the same location throughout the study period were more similar to each other than those of random species compositions, species recorded at the same point in the same study period were more different from each other than expected by chance. This effect was relatively weak during the early survey, when the observed birds had significantly higher mean SDI values than only 26.3% of the randomly generated species communities, but during the late survey, the bird communities observed at the same point had significantly higher mean SDI values than all of the randomly generated communities. This suggests that while there is no evidence for spatial partitioning of the signal space throughout the whole breeding season, there appears to be seasonal spatial signal space partitioning, particularly strong in species active later in the breeding season. Białowieża Forest provides multiple microhabitat types that support different species assemblages of generalist and specialist breeders (Wesołowski et al. 2010). Furthermore, the singing species composition in temperate regions changes over the season, as migrant birds arrive to breed at different times, while early-laying species may lay multiple clutches over the breeding period (Wesołowski and Cholewa 2009; Dunn and Møller 2014), which can increase the pressure on acoustic space use and lead to niche partitioning.

Surprisingly, while we observed seasonal spatial signal space partitioning in the entire forest bird community vocalizing throughout the day, the species active during the same minute had songs significantly more similar to each other compared to random species assemblages from the same location. This trend was not constant throughout the day, as the birds active in the 2 h before sunrise were either not different from random (early survey) or had moderately higher SDI (late survey) than the null model species communities from that time period, while after sunrise the birds observed in both parts of the season had significantly lower SDI than the null model communities from species present at the location in their respective 2-h time periods.

The presence of daylight, and different light levels due to developing foliage may contribute to this shift, as in the absence of light, the animals need to rely on sound, making auditory information more important and increasing the pressure on signal space overlap avoidance. After sunrise, the birds can supplement their communication with visual signals (Bradbury and Vehrencamp 2011; Sirkiä and Qvarnström 2021), thus reducing the pressure on acoustic space.

The shift in song dissimilarity may also stem from the number and ecology of species active at different times of the day. In the 2 h before sunrise, the number of species singing in the same minute reached four on average, and frequently included species considered primarily nocturnal (e.g., Eurasian woodcock, European nightjar, or tawny owl *Strix aluco*), as well as common species considered to be diurnal or crepuscular (e.g., common blackbird, European robin, or song thrush). The nocturnal species were frequently the only members of their genus or family included in the study, with evolutionary divergent songs that had high interspecific SDI values with many of the other species.

The peak of vocal activity in both surveys occurred just after sunrise, with over five species recorded in the same minute on average, corresponding to the peak of the dawn chorus (Staicer et al. 1996). Dawn chorus usually occurs just before the environmental conditions are optimal to begin foraging (McNamara et al. 1987), and is characterized by high singing activity of multiple birds. After sunrise, the species singing in the same minute in Białowieża Forest had songs more similar to each other than expected by chance, with the mean interspecific SDI decreasing throughout the day together with the decreasing number of singing species. The common diurnal species, including thrushes, flycatchers, and the common chaffinch, were present at all recording points throughout the daylight hours, and had relatively low interspecific SDI values with many other diurnal species (see Figure 1). Coordinated singing of acoustically similar species has been reported previously at dusk in a temperate forest by Malavasi and Farina (2013), who determined that although the concurrently singing species had similar frequency range, the real vocalization spectral overlaps were lower than expected by chance, thus avoiding signal jamming.

In this study, we did not analyze small-scale temporal partitioning within the 1-min recording samples, or the signal plasticity of the vocalizing species, but such acoustic space competition avoidance mechanisms have been reported in previous studies. Small-scale temporal vocalization pattern shifts have been reported to avoid signal jamming between two North American songbirds (Ficken et al. 1974), while veeries *Catharus fuscesnes,* which were masked by multiple species during the dawn chorus, were found to also sing at dusk when competition was reduced (Belinsky et al. 2012). Temporal, rather than spatial partitioning of signal space has also been reported in some tropical bird communities in Costa Rica and Hawaii (Hart et al. 2021), while both spatial and temporal partitioning was observed in the Neotropical bird community of sedentary species in Brazil (Luther 2009).

Because the SDI values are the result of comparisons of every reference song to every other reference song, rather than direct comparisons of overlapping songs on the dawn chorus recordings, they may not reflect the true spectral similarity of each pair of observed vocalizations, particularly in case of species with large repertoires of highly variable, complex songs. Additionally, the distance to the recorder and amplitude were not measured when calculating the SDI and detecting birds on survey recordings. As increased amplitude can mitigate masking interference of a signal (Brumm 2004; Nemeth et al. 2013), the spectrally similar sounds occurring at the same location may not interfere with each other due to relative amplitude differences. Future studies controlling for sound amplitude and directly examining the temporal and spectral responses to competing vocalizations are needed to determine the fine-scale acoustic space competition avoidance mechanisms of individual species.

Furthermore, care must be taken to provide enough high-quality reference recordings, which have no background noise and reflect the full repertoire of each species. We collected 15% of our reference recordings in Białowieża Forest, however the remaining files were downloaded from the Xeno-canto birdsong database and included recordings from other locations in Poland, and when those were not available, from elsewhere in Europe. While it is possible that regional song dialects could affect the SDI values, the larger dataset created from all available recordings provided a more stable index (Bradfer-Lawrence et al. 2019).

We based the SDI on the Acoustic Dissimilarity Index, which has been broadly applied to whole soundscape recordings, for example, in monitoring bird community changes (Sueur, Pavoine, et al. 2008; Depraetere et al. 2012; Gasc et al. 2013; Lellouch et al. 2014). Here, we demonstrate its novel use in behavioral ecology studies, providing a tool for examining interspecific competition on multiple scales, and revealing different mechanisms of species interactions. We demonstrated that birds species in a temperate forest use seasonal spatial signal space partitioning and that daily temporal niche partitioning may only occur at certain times before sunrise, with no evidence of large-scale temporal partitioning in the daytime.

## Supplementary Material

arad075_suppl_Supplementary_Material

## Data Availability

Analyses reported in this article can be reproduced using the data provided by Staniewicz et al (2023).

## Notes

We are grateful to the Białowieża Forest District for help with fieldwork organization. The study was conducted under permission of Regional Director of Environment Protection in Białystok (WPN.6205.5.2021.MM).
